# “*Candidatus* Desulfobulbus rimicarensis,” an Uncultivated Deltaproteobacterial Epibiont from the Deep-Sea Hydrothermal Vent Shrimp *Rimicaris exoculata*

**DOI:** 10.1128/AEM.02549-19

**Published:** 2020-04-01

**Authors:** Lijing Jiang, Xuewen Liu, Chunming Dong, Zhaobin Huang, Marie-Anne Cambon-Bonavita, Karine Alain, Li Gu, Shasha Wang, Zongze Shao

**Affiliations:** a Key Laboratory of Marine Genetic Resources, Third Institute of Oceanography, Ministry of Natural Resources, Xiamen, China; b State Key Laboratory Breeding Base of Marine Genetic Resources, Xiamen, China; c Fujian Key Laboratory of Marine Genetic Resources, Xiamen, China; d LIA1211, Sino-French Laboratory of Deep-Sea Microbiology (MICROBSEA), Xiamen-Plouzané, Xiamen, China; e Ifremer, Université de Brest, CNRS, Laboratoire de Microbiologie des Environnements Extrêmes LM2E, Plouzané, France; Chinese Academy of Sciences

**Keywords:** *Deltaproteobacteria*, *Rimicaris exoculata*, Wood-Ljungdahl pathway, epibiont, sulfur disproportionation

## Abstract

The deep-sea hydrothermal vent shrimp Rimicaris exoculata largely depends on a dense epibiotic chemoautotrophic bacterial community within its enlarged cephalothoracic chamber. However, our understanding of shrimp-bacterium interactions is limited. In this report, we focused on the deltaproteobacterial epibiont of R. exoculata from the relatively unexplored South Mid-Atlantic Ridge. A nearly complete genome of a Deltaproteobacteria epibiont was binned from the assembled metagenome. Whole-genome phylogenetic analysis reveals that it is affiliated with the genus *Desulfobulbus*, representing a potential novel species for which the name “*Candidatus* Desulfobulbus rimicarensis” is proposed. Genomic and transcriptomic analyses reveal that this bacterium utilizes the Wood-Ljungdahl pathway for carbon assimilation and harvests energy via sulfur disproportionation, which is significantly different from other shrimp epibionts. Additionally, this epibiont has putative nitrogen fixation activity, but it is extremely active in directly taking up ammonia and urea from the host or vent environments. Moreover, the epibiont could be distinguished from its free-living relatives by various features, such as the lack of chemotaxis and motility traits, a dramatic reduction in biosynthesis genes for capsular and extracellular polysaccharides, enrichment of genes required for carbon fixation and sulfur metabolism, and resistance to environmental toxins. Our study highlights the unique role and symbiotic adaptation of Deltaproteobacteria in deep-sea hydrothermal vent shrimps.

**IMPORTANCE** The shrimp *Rimicaris exoculata* represents the dominant faunal biomass at many deep-sea hydrothermal vent ecosystems along the Mid-Atlantic Ridge. This organism harbors dense bacterial epibiont communities in its enlarged cephalothoracic chamber that play an important nutritional role. Deltaproteobacteria are ubiquitous in epibiotic communities of *R. exoculata*, and their functional roles as epibionts are based solely on the presence of functional genes. Here, we describe “*Candidatus* Desulfobulbus rimicarensis,” an uncultivated deltaproteobacterial epibiont. Compared to campylobacterial and gammaproteobacterial epibionts of *R. exoculata*, this bacterium possessed unique metabolic pathways, such as the Wood-Ljungdahl pathway, as well as sulfur disproportionation and nitrogen fixation pathways. Furthermore, this epibiont can be distinguished from closely related free-living *Desulfobulbus* strains by its reduced genetic content and potential loss of functions, suggesting unique adaptations to the shrimp host. This study is a genomic and transcriptomic analysis of a deltaproteobacterial epibiont and largely expands the understanding of its metabolism and adaptation to the *R. exoculata* host.

## Abstract (precis)

The shrimp *Rimicaris exoculata* represents the dominant faunal biomass at many deep-sea hydrothermal vent ecosystems along the Mid-Atlantic Ridge. This organism harbors dense bacterial epibiont communities in its enlarged cephalothoracic chamber that play an important nutritional role. Deltaproteobacteria are ubiquitous in epibiotic communities of *R. exoculata*, and their functional roles as epibionts are based solely on the presence of functional genes. Here, we describe “*Candidatus* Desulfobulbus rimicarensis,” an uncultivated deltaproteobacterial epibiont. Compared to campylobacterial and gammaproteobacterial epibionts of *R. exoculata*, this bacterium possessed unique metabolic pathways, such as the Wood-Ljungdahl pathway, as well as sulfur disproportionation and nitrogen fixation pathways. Furthermore, this epibiont can be distinguished from closely related free-living *Desulfobulbus* strains by its reduced genetic content and potential loss of functions, suggesting unique adaptations to the shrimp host. This study is a genomic and transcriptomic analysis of a deltaproteobacterial epibiont and largely expands the understanding of its metabolism and adaptation to the *R. exoculata* host.

## INTRODUCTION

The shrimp Rimicaris exoculata (1) dominates the macrofauna at many hydrothermal vent sites along the Mid-Atlantic Ridge (MAR), aggregating around active hydrothermal vent chimneys in the mixing zone between electron-donor-rich hydrothermal fluids and the surrounding cold oxygenated seawater. Densities of up to 3,000 individuals per m^2^ have been observed (2). R. exoculata harbors high concentrations of epibiotic bacteria on the inner side of the enlarged cephalothoracic chamber and modified mouthparts, highlighting a symbiosis between the shrimp and its epibionts (3–6). A number of studies have focused on the nature of this association and its benefits for the shrimp, which have suggested that the shrimp obtains organic matter mainly from epibiotic bacteria that inhabit the cephalothoracic chamber rather than by grazing on free-living bacteria that are associated with chimney walls (5, 7–9). Furthermore, both inorganic carbon fixation by these chemosynthetic epibionts and transtegumental absorption of dissolved organic matter from epibionts to the shrimp have been demonstrated using isotope-labeling experiments (10).

Recent studies have demonstrated that *R. exoculata* epibiotic communities consist of a large diversity of *Campylobacteria* (previously *Epsilonproteobacteria*), *Gammaproteobacteria*, Deltaproteobacteria, *Alphaproteobacteria*, *Zetaproteobacteria*, *Betaproteobacteria*, and *Bacteroidetes* (6, 11–13). The growth of the epibiotic chemolithoautotrophs can be driven by a variety of electron sources, such as reduced sulfur compounds, molecular hydrogen, methane, and iron (5, 6, 11–13). Based on a functional gene survey, two carbon fixation pathways have been highlighted in the epibiotic communities in *R. exoculata* cephalothoracic chambers, namely, the reductive tricarboxylic acid (rTCA) cycle and the Calvin-Benson-Bassham (CBB) cycle (11). A recent metagenomic study performed on a shrimp from the Rainbow hydrothermal vent field revealed that the rTCA and CBB cycles were used for carbon fixation by two filamentous epibionts belonging to the *Campylobacteria* and the *Gammaproteobacteria*, respectively. These epibionts could couple the oxidation of reduced sulfur compounds or molecular hydrogen to oxygen or nitrate reduction (13). In addition, synthetic products from epibiotic chemoautotrophy, such as amino acids, sugars, and vitamins, could be transferred to the shrimp (13).

Meta-omics methods are very useful for adequately identifying and investigating epibiont genetic potential, as most symbiotic bacteria are resistant to *in vitro* cultivation. An early report on *R. exoculata* epibionts provided the first insights into potential metabolisms of the epibionts, based on three genomic bins belonging to *Gammaproteobacteria*, *Campylobacteria*, and *Zetaproteobacteria* (13). However, these three genome sequences were incomplete, and their complete metabolic relationships could not be reconstructed, thereby preventing the interactions with the shrimp host to be predicted. In addition to *Campylobacteria* and *Gammaproteobacteria*, Deltaproteobacteria are also frequently detected in epibiotic communities of *R. exoculata* from different deep-sea hydrothermal sites, as revealed by 16S rRNA gene sequencing, fluorescence *in situ* hybridization (FISH), and metagenomic analysis (11–13). For example, Deltaproteobacteria were highly represented in clone libraries of shrimp epibionts from the Snake Pit hydrothermal vent field (11) and were present in nearly all life stages of the shrimp at the Logachev vent site (12). These studies tend to indicate that Deltaproteobacteria might play a role in shrimp-epibiont interactions. Moreover, Hugler et al. proposed that these epibionts could perform sulfate reduction or sulfur disproportionation only based on the presence of functional genes: the *aprA* gene coding for 5′-adenylylsulfate reductase and the *hynL* gene encoding the large subunit of a [NiFe] hydrogenase (11). Therefore, the ecological functions and potential benefits for the shrimp host remain poorly understood so far, largely due to a lack of genome-level investigations.

In this study, we investigated the Deltaproteobacteria associated with the cephalothoracic chamber of shrimp sampled from a new hydrothermal vent field, named “Deyin,” in the South Mid-Atlantic Ridge (SMAR). Using integrated metagenomics and metatranscriptomics, we assembled and binned the genome of a novel species called “*Candidatus* Desulfobulbus rimicarensis,” which represents a draft genome of an uncultivated deltaproteobacterial epibiont of a deep-sea hydrothermal vent shrimp. Next, we investigated the evolutionary relationships, metabolic activity, and functional dissimilarity of “*Candidatus* Desulfobulbus rimicarensis” in relation to closely related free-living *Desulfobulbus* strains in order to decipher its adaptation to the shrimp host and to understand the shrimp-epibiont partnership.

## RESULTS AND DISCUSSION

### Abundance and localization of the family *Desulfobulbaceae*.

In order to assess the microbial diversity of *R. exoculata* epibionts, nine adult shrimp individuals sampled from the SMAR (see Fig. S1 in the supplemental material) were analyzed by 454 high-throughput pyrosequencing. No obvious differences in microbial community structures were observed among the individuals. The epibiotic bacteria consist mainly of *Campylobacteria*, *Gammaproteobacteria*, Deltaproteobacteria, and *Bacteroidetes* (Fig. S2) (14). Deltaproteobacteria accounted for 0.9 to 4.5% of the epibiotic community of the *R. exoculata* individuals, and *Desulfobulbaceae* accounted for 81.5% to 97.9% of the Deltaproteobacteria taxa. FISH was also performed to explore the presence of *Desulfobulbaceae* on the cephalothorax sections of the shrimp (Fig. S3). The general probe DSB706 (15), which targets most *Desulfobulbaceae* species, was used, revealing *Desulfobulbaceae* cells at the base of the setae, as previously observed in *R. exoculata* from deep-sea hydrothermal vent sites at the Mid-Atlantic Ridge (11). These Deltaproteobacteria were directly attached to the scaphognathite seta as well as near long filamentous bacteria affiliated with *Campylobacteria* or *Gammaproteobacteria*. This specific localization indicates that *Desulfobulbaceae* are not opportunistic. In addition, *Desulfobulbaceae* species were positively identified by FISH in all tested individuals (*n* = 3). In this study, a total of 16 shrimps were utilized for various analyses that were performed using 16S rRNA gene amplicon sequencing, FISH, metagenomics, and metatranscriptomics. In all 16 shrimp individuals, *Desulfobulbaceae* bacteria were found as residents of the epibiotic community of the cephalothoracic chamber, indicating that, at the SMAR hydrothermal field, these bacteria were regular epibionts in the *R. exoculata* cephalothoracic chamber.

### Genome assembly, characteristics, and phylogeny of *Desulfobulbaceae*.

*De novo* metagenomic assembly and then binning based on compositional features (tetranucleotide signatures [Fig. S4] and G+C content), followed by alignment, resulted in several genomic bins. The genomic bin affiliated with *Desulfobulbaceae*, named DR15, was chosen for further analysis. Genome completeness was estimated to be 95.65% and to have only 0.2% contamination based on the CheckM method, indicating that the draft genome had a high level of completeness. In order to determine the taxonomic position of DR15, a maximum likelihood phylogenetic tree was constructed based on 92 concatenated core genes. The result revealed that strain DR15 was affiliated with the genus *Desulfobulbus*, forming a separate branch with three metagenome-assembled genomes (MAGs) from hydrothermal venting fluids in the phylogenetic tree (Fig. S5).

**FIG 1:**
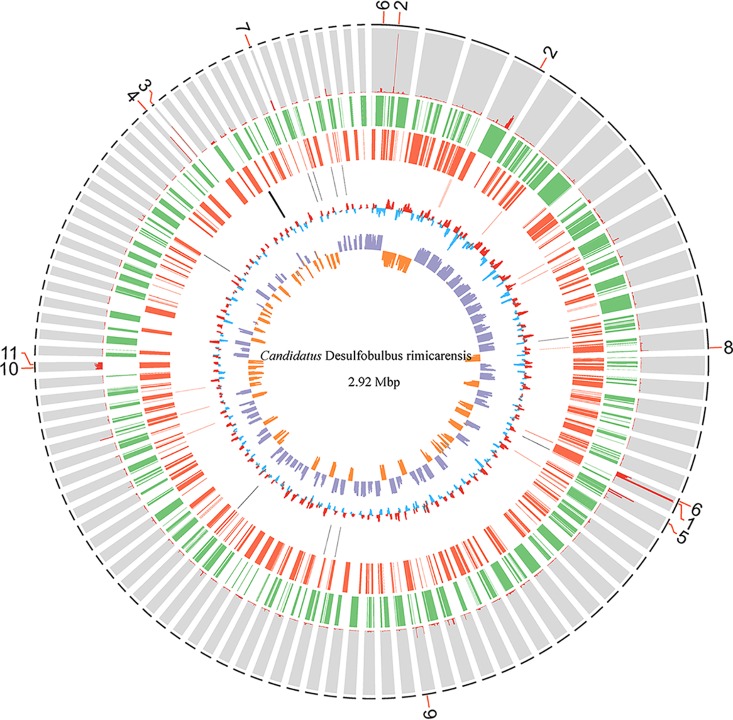
The 2.92-Mbp genome and transcriptome of “*Candidatus* Desulfobulbus rimicarensis.” The outermost ring shows the annotations of the 11 most abundant transcripts in the transcriptome. The second ring (histogram) shows the relative abundances of transcripts based on fragments per kilobase of transcript per million fragments mapped (FPKM). The third and fourth rings (green and red) indicate predicted ORFs on the plus and minus strands, respectively. The fifth ring indicates the locations of rRNA and tRNA genes. The sixth and innermost rings display the GC content and GC skew, respectively. Transcript annotations: 1, adenylylsulfate reductase (AprAB); 2, ATP synthase (AtpABCDEFFG); 3, sulfatase-modifying factor enzyme 1 (YfmG); 4, sulfate adenylyltransferase (Sat); 5, porin, hypothetical protein; 6, heterodisulfide reductase (QmoABC); 7, permease (sulfite exporter TauE/SafE); 8, TusA-related sulfurtransferase; 9, dissimilatory sulfite reductase (DsrABCD); 10, NADH-ubiquinone oxidoreductase (NuoABCDHIJKLM); 11, thiosulfate reductase (PhsAB). The FRPM of all genes in the draft genome of “*Candidatus* Desulfobulbus rimicarensis” is provided in Table S1 in the supplemental material.

The draft genome consisted of 295 contigs (2,921,535 bp long), with an average G+C content of 47.3 mol% (Fig. 1 and Table 1). The genome contained a total of 2,882 protein-coding DNA sequences, resulting in an 83.7% coding density. Approximately two-thirds (1,808) of the protein-coding genes in the genome had the highest BLAST scores against Deltaproteobacteria genomes. Of these genes, the majority (81.5%) matched against the family *Desulfobulbaceae*, and 886 coding DNA sequences had top hits with genes of *Desulfobulbus* species. Compared to the genomes of its closest free-living relatives, including Desulfobulbus mediterraneus DSM 13871, Desulfobulbus japonicus DSM 18378, and Desulfobulbus propionicus DSM 2032 (the feature summary for these 3 genomes includes sizes of 3.9 to 5.8 Mb, G+C contents of 45.8 to 58.9 mol%, and coding densities of 83.4 to 88.3%), DR15 had the smallest genome size and possessed a lower coding density than D. mediterraneus DSM 13871 and D. propionicus DSM 2032 (Table 1). The genomic size of DR15 was reduced 24 to 50% compared to the most closely related strains. The genome of DR15 had low values of average nucleotide identity (ANI) compared with the genomes of its closest relatives; the highest match was with *D. mediterraneus* DSM 13871, with 66.77% ANI, followed by D. japonicus DSM 18378 (66.62%) and *D. propionicus* DSM 2032 (66.53%) (Table 1). These associations are all far below the threshold ANI value of 94 to 96% for species delineation (16), suggesting that strain DR15 represents a novel species.

**TABLE 1:** General genomic features of “*Candidatus* Desulfobulbus rimicarensis” and its closest free-living relatives

Parameter	Value for organism			
	“*Candidatus* Desulfobulbus rimicarensis DR15”	Desulfobulbus mediterraneus DSM 13871	Desulfobulbus japonicus DSM 18378	Desulfobulbus propionicus DSM 2032
Completeness	Draft	Draft	Draft	Complete
ANI (%)		66.77	66.62	66.53
Database and accession no.	BioProject PRJNA479708	GenBank AUCW01000000	GenBank AUCV01000000	GenBank CP002364
Genome size (bp)	2,921,535	4,784,586	5,794,886	3,851,869
GC content (%)	47.3	57.6	45.8	58.9
No. of protein-coding genes	2,882	3,819	4,802	3,255
Coding density (%)	83.7	85.4	83.4	88.3
Isolation source	Hydrothermal vent shrimp	Deep-sea sediment	Estuarine sediment	Freshwater mud

Combining the above-described data, we propose that DR15 should be assigned as a novel species of the genus *Desulfobulbus*, named “*Candidatus* Desulfobulbus rimicarensis.” Strain DR15 is a deltaproteobacterial representative of the epibionts of this deep-sea hydrothermal vent shrimp.

### Metabolism.

Integrated metagenomic and metatranscriptomic analyses were used to decipher the metabolic potential and transcriptional activity (RNA expression) of “*Candidatus* Desulfobulbus rimicarensis.” Once onboard, live shrimps were immediately frozen in liquid nitrogen. Although we cannot exclude that the expression of the mRNAs may have been partially modified during the sample ascent, we have nevertheless an approximation of expression *in situ*.

### Carbon fixation and central carbon metabolism.

In contrast to the epibiotic chemolithoautotrophs previously described in *R. exoculata*, strain DR15 probably uses the Wood-Ljungdahl (WL) pathway for carbon fixation. The epibiont genome contains nearly the complete set of genes required for the WL pathway, including homologs of formate dehydrogenase (*fdhA*, *fdhD*, and *fdhF*), formyl-tetrahydrofolate (THF) synthetase (*fhs*), methylene-THF dehydrogenase (*folD*), methylene-THF reductase (*metF*), methyltransferase (*acsE*), bifunctional carbon monoxide dehydrogenase/acetyl-CoA synthase (*acsABC*), phosphotransacetylase (*pta*), and acetate kinase (Fig. S6 and Table S1) genes. The *fchA* gene encoding the formyl-THF cyclohydrolase, which is responsible for converting formyl-THF into methyl-THF, was absent in the draft genome. Previous studies suggest that this gene is not essential for the WL pathway (17, 18). The *metV* gene was also absent; instead, two copies of the *metF* gene were found in strain DR15, which could possibly replace the role of MetV in catalyzing the methylenetetrahydrofolate reductase reaction. This agrees with data from previous studies of Acetohalobium arabaticum (18), Thermus thermophilus (19), and Escherichia coli (20). In addition, the epibiont genome contained genes encoding THF biosynthesis, corrinoid iron-sulfur protein, and molybdopterin cofactor, which play key roles in single-carbon transfer for synthesizing acetyl-CoA from carbon dioxide and molecular hydrogen (21). This suggests that strain DR15 could synthesize these cofactors to meet the requirements of the WL pathway. In addition, the strain DR15 genome possessed nearly all of the genes needed to reconstruct the complete central pathways, such as the TCA cycle as well as the Embden-Meyerhof-Parnas, pentose phosphate, gluconeogenesis, and methylmalonyl-CoA pathways (see Fig. 3 and Table S1).

All genes required for carbon fixation and central carbon metabolism described above were found to be actively transcribed in strain DR15 among the studied samples (Table S1). The genes *acsE*, *fhs*, and *fdhF* for the WL pathway; *gltA* for citrate synthase in the TCA cycle; *talA* encoding a transaldolase associated with the pentose phosphate pathway; *actP* for acetate transport; and *porA* for the conversion of acetyl-CoA to pyruvate had the highest transcript abundances (see Fig. 3 and Table S1). These data indicated that carbon fixation, acetate uptake, the TCA cycle, and the pentose phosphate pathway were active in strain DR15, and around 98 to 99% of the acetyl-CoA synthesized via the WL pathway could be converted into pyruvate, which links the autotrophic WL pathway to heterotrophic metabolism. In addition, a carbonic anhydrase-encoding gene, functioning as a carbon dioxide concentrator that elevates inorganic carbon levels for fixation, was highly expressed. These results suggest that this epibiont could be an active chemoautotroph growing by using the WL pathway for carbon fixation.

The WL pathway was the only carbon fixation pathway discovered in this bacterium. Previous studies have revealed two other carbon fixation pathways, the rTCA cycle and the CBB cycle, from the epibiotic chemolithoautotrophs of the same vent shrimp species collected further north of the Mid-Atlantic Ridge (13), in epibionts belonging to *Campylobacteria* and *Gammaproteobacteria*. We identified a bacterial symbiont from a vent animal host that is likely to use the WL pathway for carbon fixation. We also report a carbon fixation pathway in a member of the genus *Desulfobulbus*. The pathway has been highlighted in the sulfur-disproportionating bacterium Desulfocapsa sulfexigens (22), which is closely related to strain DR15. In addition, most of the enzymes in the WL pathway encoded in the genome of strain DR15 were most closely related to members of the Deltaproteobacteria (Fig. S7 and S8). We propose that as a primary producer in the epibiotic community, the WL pathway could compensate for the rTCA and CBB pathways and could support the growth of the dominant vent fauna.

### Disproportionation of inorganic sulfur compounds.

The biological disproportionation of inorganic sulfur compounds is a microbiologically catalyzed chemolithotrophic process in which sulfur compounds, such as elemental sulfur, thiosulfate, and sulfite, serve as both electron donors and acceptors in order to generate hydrogen sulfide and sulfate. The microbes involved in this type of “inorganic fermentation” or “mineral fermentation” are phylogenetically related to several phyla: *Thermodesulfobacteria* (23, 24), *Firmicutes* (25), *Gammaproteobacteria* (26), and Deltaproteobacteria (27–32). The latter is generally regarded as a lineage of sulfate reducers (29). Moreover, the capacity to disproportionate inorganic sulfur compounds is relatively common among sulfate reducers (29). In this study, we hypothesize that “*Candidatus* Desulfobulbus rimicarensis” grew via the disproportionation of reduced sulfur compounds such as thiosulfate, sulfite, and elemental sulfur (see Fig. 3 and Table S1).

**FIG 2:**
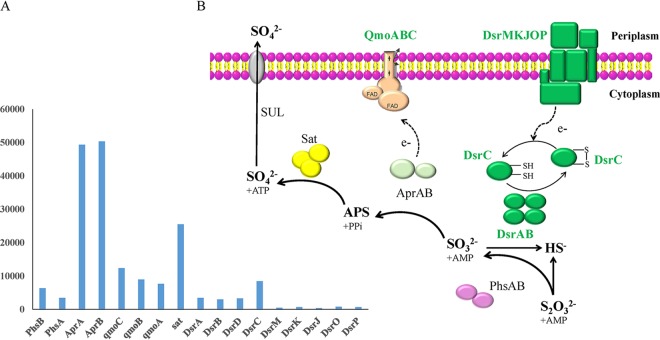
(A) Transcript abundances of key genes involved in the disproportionation of inorganic sulfur compounds in “*Candidatus* Desulfobulbus rimicarensis.” (B) Disproportionation of inorganic sulfur compounds and energy conservation in “*Candidatus* Desulfobulbus rimicarensis.” Transcript abundance is normalized for gene length and the total number of reads per data set (FPKM). Abbreviations: AprAB, adenylylsulfate reductase; Sat, sulfuradenylyltransferase; DsrABCD, reverse-type dissimilatory sulfite reductase; PhsAB, thiosulfate reductase; DsrMKJOP, sulfite reduction-associated complex; QmoABC, putative quinone-interacting membrane-bound oxidoreductase; SUL, sulfate permease; APS, adenylyl sulfate.

Two thiosulfate reductases (encoded by *phsAB*) were found in the strain DR15 genome and could catalyze the initial step of thiosulfate disproportionation, probably by converting thiosulfate into sulfite and hydrogen sulfide (or, less likely, to sulfite and elemental sulfur) (33, 34). Thereafter, there are two parallel ways for the oxidation of sulfite to sulfate reported in the literature: (i) the sulfate reduction pathway in the reverse direction and (ii) the activity of sulfite oxidoreductase (29, 33). The strain DR15 genome contains the complete pathway for dissimilatory sulfate reduction, including ATP sulfurylase (encoded by the gene *sat*), APS reductase (gene *aprAB*), and dissimilatory sulfite reductase (gene *dsrABCD*). Also, genes encoding the adenylylsulfate reductase (APS) reductase-associated electron transfer complex (QmoABC) and dissimilatory sulfite reductase-associated electron transport proteins (DsrMKJOP) are present in this genome (Fig. 2B). However, there are no genes that code for sulfite oxidoreductase (Table S1), indicating that strain DR15 likely uses the reverse sulfate reduction pathway to oxidize sulfite to sulfate during thiosulfate disproportionation. The disproportionation of elemental sulfur can also occur via this route, although the first step differs from that of thiosulfate disproportionation and is not well described. The capacity to couple growth to the disproportionation of thiosulfate or elemental sulfur has been observed in Desulfobulbus propionicus (29, 35), a close relative to the epibiont within the *Desulfobulbaceae* family (Fig. S5). In addition, the predominance of *Desulfobulbaceae* members has also been demonstrated in elemental sulfur-disproportionating enrichment cultures (29). Thus, we propose that strain DR15 may be capable of inorganic sulfur compound disproportionation. Furthermore, transcriptomic analysis revealed that all of the genes involved in the disproportionation of reduced sulfur compounds were expressed (Fig. 2A and Table S1). The genes *aprAB* for APS reductase and *sat* encoding ATP sulfurylase had the highest abundances among all transcripts, followed by *phsAB* for thiosulfate reductase and *dsrABCD* for dissimilatory sulfite reductase. These data, including the expression of *phsAB* genes that are not expressed during sulfate reduction, confirmed that the disproportionation of inorganic sulfur compounds was active. Therefore, it is likely that thiosulfate disproportionation provides energy for the growth of strain DR15. However, it is also possible that the epibiont might grow via sulfate reduction under certain conditions.

Previously, the growth of the epibiotic chemolithoautotrophs associated with *R. exoculata* and affiliated with *Gammaproteobacteria*, *Campylobacteria*, *Alphaproteobacteria*, and *Zetaproteobacteria* was found to be fueled by the oxidation of reduced sulfur compounds, molecular hydrogen, methane, and iron (5, 6, 11–13). This study demonstrates that chemoautotrophic epibionts of *R. exoculata* are likely to be powered by the disproportionation of inorganic sulfur compounds. Hugler et al. had previously made this assumption based on the detection of *aprA* sequences from Deltaproteobacteria during molecular screening of functional genes (11). In the cephalothoracic chamber, energy production through sulfur compound disproportionation would prevent competition with cooccurring epibionts for energy sources.

### Hydrogen oxidation.

Genomic analysis revealed that the DR15 genome encoded four [NiFe] hydrogenases, two periplasmic group 1 hydrogenases (Hya and Hyb); one cytoplasmic, methyl-viologen-reducing hydrogenase (Mvh); and one membrane-associated energy-converting [NiFe] hydrogenase (Ech) (Fig. S9 and Table S1), while no [FeFe] hydrogenase genes were detected in the draft genome. Group 1 [NiFe] hydrogenases are membrane-bound respiratory hydrogenases performing hydrogen oxidation linked to quinone reduction (36). Mvh hydrogenases are usually associated with heterodisulfide reductases (Hdr) as large complexes (MvhADG/HdrABC), which are proposed to couple the endergonic reduction of ferredoxin with molecular hydrogen to the exergonic reduction of the heterodisulfide with molecular hydrogen by electron bifurcation (37, 38). In addition, the *mvhADG* genes are sometimes physically located next to *hdr* genes in some sulfate-reducing organisms and can act as electron acceptors in a process that may involve electron bifurcation (39). In the DR15 genome, the *mvhADG* genes were also adjacent to the *hdr* genes (Fig. S6 and Table S1), indicating that the Mvh hydrogenase may perform the same function as in sulfate-reducing bacteria. Ech complexes are widespread in both anaerobic and facultative anaerobic bacteria/archaea and couple exergonic electron transfer from reduced ferredoxin to H^+^ or the reduction of ferredoxin with molecular hydrogen (21). In the epibiont genome, the gene cluster encoding the Ech complex was present in the same synton as a gene coding for a putative formate dehydrogenase (Table S1). Therefore, the Ech complex is a possible candidate for energy coupling in the WL pathway of strain DR15 (Fig. S6). This is similar to Moorella thermoacetica, in which Ech activity is coupled to the generation of a transmembrane electrochemical H^+^ gradient (21).

Transcriptomic analysis revealed that all of these hydrogenase genes were expressed. They were involved either in the oxidation of H_2_ coupled to the reduction of sulfate or in electron transfer and cofactor regeneration. We observed that, at the time of sampling, genes encoding hydrogenases were expressed at significantly lower levels than genes involved in the disproportionation of inorganic sulfur compounds (Table S1). Hence, based on the transcriptomic data, at the time of sampling, the strain might harvest more energy from sulfur compound disproportionation than from hydrogen oxidation.

### Nitrogen metabolism.

**FIG 3:**
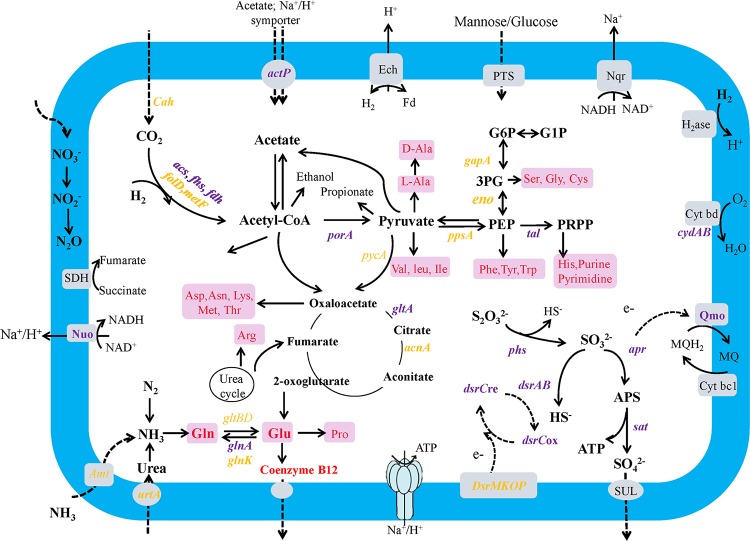
Metabolic map reconstructed from the draft genome of “*Candidatus* Desulfobulbus rimicarensis.” Biosynthetic amino acids and central vitamins and cofactors are indicated in red on a pink background. Gene transcripts that are highly expressed are emphasized in purple, and genes with moderate transcript abundances are indicated in orange. Abbreviations: G1P, glucose 1-phosphate; G6P, glucose 6-phosphate; PRPP, phosphoribosyl pyrophosphate; PEP, phosphoenolpyruvate; PG, phosphoglycerate; Ala, alanine; Arg, arginine; Asn, asparagine; Asp, aspartate; Gln, glutamine; Glu, glutamate; Gly, glycine; Ile, isoleucine; Leu, leucine; Lys, lysine; Phe, phenylalanine; Pro, proline; Ser, serine; Thr, threonine; Trp, tryptophan; Tyr, tyrosine; Val, valine; His, histidine; Met, methionine; Cys, cysteine; Cah, carbonic anhydrase; PorA, pyruvate-flavodoxin oxidoreductase; PpsA, phosphoenolpyruvate synthase; Tal, transaldolase; Eno, enolase; GapA, glyceraldehyde-3-phosphate dehydrogenase; PycA, pyruvate carboxylase; GltA, citrate synthase; AcnA, aconitate hydratase; Amt, ammonium transporter; Urt, urea ABC transporter; GlnA, glutamine synthetase; GlnK, NifHD-nitrogen regulatory protein P-II; Glt, glutamate synthase; ActP, acetate permease; PTS, phosphotransferase system; Nuo, NADH-ubiquinone oxidoreductase; DmsE, decaheme *c*-type cytochrome; CydAB, cytochrome *d* ubiquinol oxidase; SDH, succinate dehydrogenase; Cyt bc1, cytochrome *bc*_1_-type ubiquinol oxidoreductase; Cyt bd, *bd*-type cytochrome oxidase. For genes present in sulfur disproportionation and WL pathways, see Fig. 2 and Fig. S6 in the supplemental material.

Based on the genomic data, DR15 has the potential to use ammonia, urea, and molecular nitrogen as nitrogen sources, which represent a wider range of nitrogen sources than previously described for campylobacterial and gammaproteobacterial epibionts of *R. exoculata* (13). The draft genome contains genes encoding ammonium permeases (Amt), glutamine synthase (GlnA), and glutamate synthase (GltBD) for ammonia assimilation (Fig. 3 and Table S1). The *glnK* gene encoding a regulatory protein, P-II, was linked to the *amt* gene for ammonia transport, indicating that nitrogen metabolism in DR15 could be regulated similarly to that in E. coli (40). In addition, the DR15 genome encodes a urea ABC transporter for urea uptake as well as a urease operon that is involved in urea hydrolysis, suggesting that DR15 could also utilize urea to generate ammonia, which has not been observed in the campylobacterial and gammaproteobacterial epibionts (13). Surprisingly, DR15 was found to potentially be capable of nitrogen fixation. Nearly all of the genes involved in this process were present within the draft genome, including *nifHDK* encoding a molybdenum-iron nitrogenase; *nifENB*, *nifU*, and *nifS* for assembly proteins; and *nifA* and *ntrXY* for regulatory proteins (Table S1). Similarly, in the sulfur-disproportionating deltaproteobacterium Desulfocapsa sulfexigens, all of the genes necessary for nitrogen fixation were observed in the genome (22). Therefore, it is possible that DR15 can grow by utilizing free nitrogen gas as the sole nitrogen source. Symbiotic nitrogen fixers are known to be associated with wood-boring bivalves, coral, sponges, and sea urchins (41). Recently, Petersen et al. provided the first report of nitrogen fixation by a chemosynthetic symbiont in a shallow-water bivalve (42). Nitrogen fixation may be more important in the deep-sea environment, especially as nitrogen sources are scarce. However, prior to this study, nitrogen fixation pathways had not been detected in vent animal symbionts. This study reports nitrogen fixation in a chemosynthetic epibiont of *R. exoculata*. In addition, the presence of one denitrification system, including the periplasmic dissimilatory nitrate reductase (Nap) and the nitrite reductase (Nir), indicated that DR15 might have the potential to reduce nitrate to nitrous oxide by dissimilatory nitrate reduction. This ability was also discovered in a gammaproteobacterial epibiont of *R. exoculata* (13).

Transcriptomic data revealed that almost all of the genes required for nitrogen metabolism described above were also expressed in strain DR15 (Fig. 3 and Table S1). Among these, genes involved in ammonia assimilation, such as *amt*, *glnA*, *gltBD*, and *glnK*, showed the highest levels of expression, followed by the *urtA* gene for a urea ABC transporter (Fig. 3). In contrast, the genes involved in nitrogen fixation were expressed at relatively low levels. These results indicated that, at the time of sampling, DR15 might utilize ammonia as the main nitrogen source, followed by urea. Moreover, ammonia was assimilated mainly by glutamine synthase (Fig. 3). Nitrogen fixation may be active when the environment is depleted of nitrogen sources such as ammonia and urea.

### Oxidative stress.

The DR15 genome contains multiple copies of genes involved in defense against oxidative stress, such as the rubrerythrin (Rbr)-rubredoxin (Rbo) oxidoreductase system (Table S1). This system consists of Rbr and Rbo, which have been proposed in Desulfovibrio vulgaris to be an oxidative stress protection system that is an alternative to superoxide dismutase (SOD) (43). In addition, the genome also encodes a *bd*-type cytochrome terminal oxidase (Fig. 3 and Table S1). This enzyme reduces molecular oxygen using electrons from the quinone pool in *Desulfovibrio* species (44), thereby protecting cells from molecular oxygen. The presence and expression of the Rbr-Rbo system and of its regulator (PerR), as well as *bd*-type cytochromes, could indicate that “*Candidatus* Desulfobulbus rimicarensis” encounters a wide range of redox gradients as the shrimp swims through the vent environment.

### Amino acid and cofactor biosynthesis.

Strain DR15 can synthesize all 20 amino acids, as all the genes essential for amino acid biosynthesis were present in the genome and were expressed (Table S1). DR15 also contains all genes required for the biosynthesis of selenocysteine, an essential catalytic component of the selenium-containing variant of formate dehydrogenase in the WL pathway (18). In addition, this bacterium has the genetic potential to synthesize vitamins B_12_, B_1_, and B_6_ as well as many other cofactors. Vitamin B_12_ is essential for both the methyl and carboxyl branches of the WL pathway. The draft genome contains almost the complete set of genes required for synthesizing cobalamin via precorrin-2 (Table S1). The biosynthesis of biotin, heme, siroheme, riboflavin, folate, tetrahydrofolate, pantothenate, coenzyme A, NAD, NADP, and a molybdenum cofactor could also be performed in this bacterium, based on the presence of the required genes (Table S1). However, the genes involved in the biosynthesis of menaquinone are incomplete, which suggests that the epibiont might depend on an external supply of this compound or that these genes were not captured due to missing portions of the draft genome. All genes involved in cofactor biosynthesis were expressed, and the genes required for vitamin B_6_ biosynthesis exhibited the highest expression levels.

### Comparative genomic analyses suggest adaptations to an epibiotic lifestyle.

**FIG 4:**
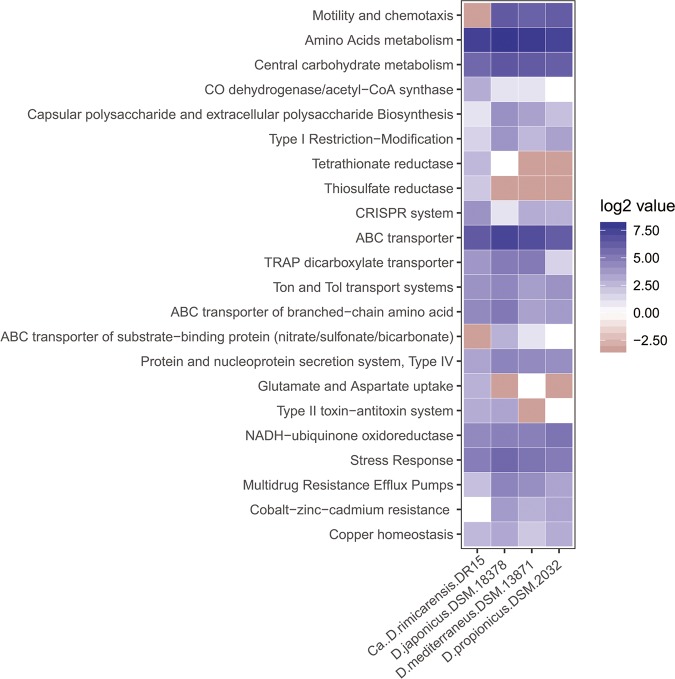
Major differential genes between “*Candidatus* Desulfobulbus rimicarensis” and closely related free-living *Desulfobulbus* strains based on bacterial pangenome analysis.

A comparative whole-genome analysis revealed the likely adaptive features between symbiotic and free-living *Desulfobulbus* species (45–47). Genes from three *Desulfobulbus* genomes used in the comparison were subjected to pangenome analysis. Of these, 930 occurred in both the shrimp-associated and free-living genome pools, and 1,553 and 537 genes were specific for the shrimp-associated and free-living pools, respectively. As a shrimp epibiont, strain DR15 displayed unique symbiotic features, such as carbon and energy metabolisms (Fig. 4). The prediction of a functional WL pathway for carbon fixation was present only in strain DR15, with an enrichment of CO dehydrogenase/acetyl-CoA synthase (8 genes in epibiotic versus 1 or 2 genes in free-living genomes). The presence of a functional WL pathway in this epibiont might guarantee a steady carbon supply to the host and ensure its ecological success. Regarding sulfur metabolism, strain DR15 has genes that could potentially reduce tetrathionate and thiosulfate, with six genes encoding tetrathionate reductase and four genes encoding thiosulfate reductase, whereas these genes were almost completely absent in the genomes of free-living species. Moreover, seven genes encoding the uptake of glutamate and aspartate were present in the epibiont genome, whereas only one gene was found in three free-living strains, suggesting that strain DR15 may have the ability to take up glutamate or aspartate, possibly from the shrimp host, while free-living strains would not have this ability. In addition, strain DR15 also shows enrichment in CRISPR-associated proteins, including Cas, Csd, Csm, and Cmr family proteins (16 genes in strain DR15 versus 2 to 8 genes in the genomes of free-living species) (Fig. 4). The genomic signature is commonly reported as being diagnostic of a typical sponge-symbiotic lifestyle (48); here, it probably hints at an as-yet-unrevealed role of these proteins in shrimp-epibiont interactions.

The strain DR15 genome can also be distinguished from the genomes of free-living strains as it lacks genes encoding several features typically reported in free-living strains. There were no genes coding for flagellum synthesis or chemotaxis proteins in the DR15 genome, whereas around 64 to 82 genes coding for these features were present in the genomes of free-living species (Fig. 4), suggesting a nonmotile lifestyle. Genes responsible for the biosynthesis of capsular polysaccharide (CPS) and extracellular polysaccharide (EPS) were almost completely lost in strain DR15 (Fig. 4). CPS and EPS are extracellular polysaccharides common in a wide range of microorganisms that play important roles, such as in protection against environmental stresses, biofilm formation, and resistance to phagocytosis or antibiotic treatments. The absence of CPS and EPS suggests that strain DR15 is weakly protected from extracellular stresses, which is compensated by being located inside the cephalothoracic chamber. In contrast, this characteristic is likely to diminish the barrier between the symbiont and shrimp cells, thus benefiting shrimp-epibiont interactions and nutrient exchange. Furthermore, there was also a dramatic reduction in genes involved in resistance to antibiotics and environmental toxins in the strain DR15 genome (Fig. 4), such as multidrug resistance efflux and cobalt-zinc-cadmium resistance. Resistance to these toxins in open water is important for the survival of microorganisms; however, strain DR15 could escape these toxins by being sheltered by its shrimp host. In addition, the type I restriction-modification system involved in DNA metabolism as well as the type IV protein and nucleoprotein secretion system involved in membrane transport were also dramatically reduced in the epibiont genome.

### Syntrophic association.

**FIG 5:**
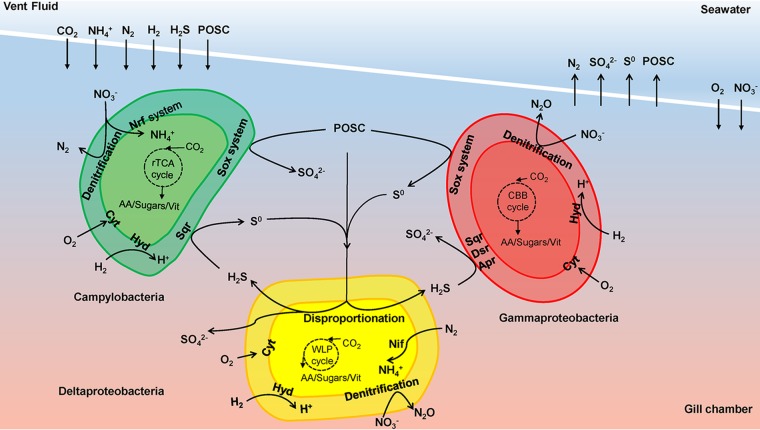
Hypothetical model of the sulfur cycle in the gill chamber of *Rimicaris exoculata* showing syntrophic cycling of oxidized and reduced sulfur compounds between sulfur-disproportionating Deltaproteobacteria epibionts and sulfur-oxidizing epibionts, including *Campylobacteria* and *Gammaproteobacteria*. Abbreviations: POSC, partially oxidized inorganic sulfur compound; Cyt, cytochrome; Hyd, hydrogenases; Sqr, sulfide-quinone oxidoreductase; Apr, adenylylsulfate reductase; Dsr, dissimilatory sulfite reductase; Nrf, cytochrome *c* nitrite reductase; Nif, nitrogenase; AA, amino acids; Vit, vitamins.

Multiple symbionts have been found to cooccur in both deep-sea and shallow-water hosts such as mussels, worms, shrimps, and snails (13, 49, 50). Stable associations between multiple symbionts within a host are assumed to be beneficial to each other (51, 52). Although the cooccurrence of sulfur oxidizers and deltaproteobacterial epibionts raises the possibility of an internal sulfur cycle that would take place within the shrimp cephalothoracic chamber (11), this hypothesis is based solely on 16S rRNA gene sequencing and functional gene surveys. This study probably supports the existence of a syntrophic relationship between sulfur-disproportionating Deltaproteobacteria and sulfur-oxidizing bacteria, including *Campylobacteria* and *Gammaproteobacteria*, which are associated with *R. exoculata* (Fig. 5). Considering that species of *Campylobacteria* and *Gammaproteobacteria* are filamentous (6, 11, 12), these bacteria anchor to the surface of the scaphognathite setae at one end of the cell and use the other end to scavenge sulfide compounds from the interior of the cephalothoracic chamber, producing partially oxidized inorganic sulfur compounds (POSCs) via sulfide oxidation. In contrast, deltaproteobacterial epibionts settle close to the surface of the scaphognathite setae, as observed by Hugler et al. (11). These bacteria seem to be able to utilize POSCs that are either (i) produced by *Campylobacteria* and *Gammaproteobacteria* or (ii) directly transferred from the surrounding environments for disproportionation. In return, the sulfide derived from disproportionation could come back to *Campylobacteria* and *Gammaproteobacteria* for reoxidation (Fig. 5). Thus, if this hypothesis was confirmed, these different epibiotic styles would not compete for energy sources but rather would share a mutualistic relationship with each other in an epibiotic sulfur cycle. These bacterial species could be specialized to fit into microniches and build a harmonious relationship with their host. Deltaproteobacterial epibionts would tend to reside in the anoxic or oxic-anoxic interfaces on the close surface of the scaphognathite setae, while *Campylobacteria* and *Gammaproteobacteria* thrive in the oxic zone, on the scaphognathite setae of *R. exoculata*. This syntrophic association would be based on the exchange of reduced and oxidized sulfur compounds, as described previously for an oligochaete worm (51).

### Description of “*Candidatus* Desulfobulbus rimicarensis.”

“*Candidatus* Desulfobulbus rimicarensis” (ri.mi′ca′ren′sis. L. fem. adj. rimicarensis, referring to *Rimicaris exoculata*, the host shrimp where the species was found; Desulfobulbus rimicarensis, a *Rimicaris exoculata* epibiont).

**(i) Properties.** Phylogenetic analysis shows that the novel strain belongs to the genus *Desulfobulbus* but forms a cluster separated from other species of this genus. The cell colonizes the surface of scaphognathite setae in the cephalothoracic chamber of *R. exoculata*, a shrimp inhabiting deep-sea hydrothermal vents from the Atlantic Ocean. The bacterium is likely to grow chemolithoautotrophically by the disproportionation of inorganic sulfur compounds, or molecular hydrogen oxidation coupled to sulfate reduction, under reducing conditions. It has the genetic potential to utilize diverse nitrogen sources, including ammonia, urea, and free nitrogen gas.

**(ii) Metabolic activity.** In its natural environment, this bacterium is likely to utilize carbon dioxide as the main carbon source. Growth might be supported by the disproportionation of reduced sulfur compounds. Ammonia and urea might be used as nitrogen sources. The metabolic plasticity and activity of this deltaproteobacterial epibiont of a shrimp are likely to confer an adaptive advantage to the shrimp in the highly dynamic hydrothermal mixing zone.

### Conclusion.

Although Deltaproteobacteria are ubiquitous in epibiotic communities of *R. exoculata* in deep-sea hydrothermal vent environments (11–13), their ecological function and symbiotic adaptation to the shrimp host are not clear. In this report, we describe a novel bacterium, “*Candidatus* Desulfobulbus rimicarensis,” which represents a characterized (while still uncultivated) deltaproteobacterial epibiont of this deep-sea hydrothermal vent shrimp. Compared to other epibionts that inhabit the cephalothoracic chamber of *R. exoculata* (13), this bacterium possesses unique metabolic pathways, such as the WL, sulfur disproportionation (and potentially also sulfate reduction), and nitrogen fixation pathways. We hypothesize that this bacterium is involved in a syntrophic association with the sulfur-oxidizing campylobacterial and gammaproteobacterial epibionts of the cephalothoracic chamber through the exchange of sulfur compounds of differing redox levels. In addition, the genome of this epibiont could be distinguished from those of its free-living counterparts by the reduced genome size, the lack of chemotaxis and motility traits, the dramatic reduction of genes involved in the biosynthesis of CPS and EPS, the lack of resistance to environmental toxins, and the enrichment of genes required for carbon fixation and sulfur metabolism. These genetic modifications suggest that “*Candidatus* Desulfobulbus rimicarensis” is adapted to its shrimp host.

## MATERIALS AND METHODS

### Shrimp collection and nucleic acid extraction.

Vent shrimps were collected using a grabber during leg III of the DY115-26 oceanographic cruise on the South MAR (site SMAR-S029-TVG11; 15.17°S, 13.36°W; 2,807-m depth) (see Fig. S1 in the supplemental material), with Zongze Shao as the chief scientist. Phylogenetic analysis of the mitochondrial cytochrome oxidase subunit I (COI) genes (14) showed that they were most closely related to the species *Rimicaris exoculata*, which was first found in the North Mid-Atlantic Ridge (1). Once aboard, *R. exoculata* specimens were immediately stored in liquid nitrogen and frozen at −80°C for DNA and RNA extractions. In the laboratory, four specimens were dissected under sterile conditions, and the mouthparts were immediately used to extract DNA and RNA. DNA was extracted using a modification of an SDS-based DNA extraction method (53). Samples were mixed with 13.5 ml DNA extraction buffer, vortexed vigorously for 1 min, and incubated in an orbital shaker at 37°C for 30 min. Next, 1.5 ml 20% SDS was added, and the samples were incubated in a shaking water bath at 65°C for 1 h. After centrifugation at 6,000 × *g* for 20 min, the DNA supernatant was precipitated with phenol, chloroform, and isopropanol. RNA was extracted using a Tri reagent procedure (54). After each extraction, DNA and RNA were assessed using a NanoDrop system (NanoDrop 2000; Thermo, Wilmington, DE, USA) and gel electrophoresis to determine concentration and integrity and then sent to the Chinese National Human Genome Center in Shanghai, China, for high-throughput sequencing.

### Assembly, binning, and annotation of the individual genome.

Metagenomic DNA sequencing was performed with the Illumina MiSeq platform (500-bp library) at the National Human Genome Center of China in Shanghai, China, according to the manufacturer’s manual. This produced a total of 14,553,576 reads, with a total length of 8.7 Gbp. All these reads were imported into CLC Genomics Workbench 6.5 (Qiagen) and trimmed using a quality score of 0.01 and a minimum length of 50 bp. Subsequently, the trimmed reads were assembled with the following modified parameters: word size of 61, bubble size of 200, and minimum contig length of 200 bp. This procedure resulted in 1,030,504 contigs (11,026 contigs of ≥1,000 bp), with a total length of 394,265,091 bp. Contig coverage was calculated by mapping the trimmed reads to a reference algorithm using a minimum similarity of 95% of the read length. Binning of the draft genomes was carried out based on tetranucleotide frequency by utilizing Databionics ESOM-map software (55) with the same parameters as the ones described previously by Dick et al. (56). Final results were manually curated for species assignment of contigs based on their coverage, GC content, and BLAST results against the nr (nonredundant) database. Contigs with ambiguous taxonomic assignments were discarded for the rest of the analysis. The purity and completeness of genome bins were then assessed by CheckM v.1.0.7 (57) using the lineage-specific workflow. Open reading frames (ORFs) were identified using Prodigal (version 2.6.3) (58). The conserved single-copy genes (CSCGs) of genome bins were identified by searching identified amino acid sequences against a hidden Markov model (HMM) database of 107 universally prokaryotic genes (59) using hmmsearch with default settings.

Gene annotation of the resulting draft genome was performed by using the Rapid Annotation Using Subsystems Technology (RAST) server (60) and the NCBI Prokaryotic Genomes Automatic Annotation Pipeline (PGAAP). Metabolic reconstruction of DR15 was performed based on a list of functional genes involved in important metabolic pathways (Table S1), each of which was automatically and then manually curated by comparing the predicted protein sequences with those in GenBank databases. In addition, the CRISPR-Cas system of strain DR15 was searched using CRISPRCasFinder (https://crisprcas.i2bc.paris-saclay.fr/CrisprCasFinder/Index). The HydDB Web server (https://services.birc.au.dk/hyddb/) was used for hydrogenase classification (61).

### Metatranscriptomic analyses.

The extracted RNA was treated with DNase I (TaKaRa, Japan) to remove genomic DNA. rRNA was removed from the total RNA using the Ribo-Zero magnetic kit (Epicentre, USA). A total of 100 ng rRNA-depleted RNA was used for cDNA library preparation. Sequencing libraries were constructed by using the NEBNext Ultra directional RNA library prep kit for Illumina (NEB, USA) according to the manufacturer’s protocol. The cDNA was directly sequenced using the Illumina HiSeq 2500 platform at the National Human Genome Center of China in Shanghai, China. The obtained raw 2- by 100-bp paired-end reads were subjected to quality control using the next-generation sequencing (NGS) FASTX-Toolkit (http://hannonlab.cshl.edu/fastx_toolkit/), with a quality score of 0.01 and a minimum length of 50 bp, resulting in a total of 5.38 Gbp of clean data. To depict the gene expression profile for each genomic bin, the dereplicated, trimmed, and paired-end Illumina reads were then mapped to contigs from the DR15 genome using Bowtie version 1.1.1 (http://bowtie-bio.sourceforge.net/index.shtml), with parameters specifically chosen for transcriptome sequencing (RNA-Seq) quantification (–n 2, –e 99999999, –l 25) (62). FPKM (fragments per kilobase of transcript per million fragments mapped) were used to estimate the expression level of each gene using RSEM-1.2.3 (http://deweylab.biostat.wisc.edu/rsem/) with default parameters.

### Phylogenetic analysis.

In order to elucidate the taxonomic positions of “*Candidatus* Desulfobulbus rimicarensis,” a bacterial core gene-based phylogenetic analysis was carried out using the Up-to-Date Bacterial Core Gene (UBCG) pipeline (63). The whole-genome sequences of reference taxa were obtained from the NCBI database. The 92 concatenated gene sequences were extracted, aligned, and concatenated within the UBCG pipeline using default parameters. A maximum likelihood phylogenetic tree was inferred using RAxML version 8.2.11 (64) with the general time reversible (GTR) model and 100 bootstrap replications.

### Comparative genomics.

Comparative genome analysis was performed using the Bacterial Pan Genome Analysis (BPGA) pipeline (65). Core genes were detected using the USEARCH program (v. 11.0) (66) and extracted from the whole-genome sequences of the four strains, with a 50% sequence identity cutoff. The pangenome analysis was also used to compile a set of accessory genes present in at least two or more strains and unique genes found in only a single strain.

### Fluorescence *in situ* hybridization.

The FISH protocol was modified based on a method described previously by Petersen et al. (6). Whole scaphognathite tissues were embedded in tissue freezing medium (Leica, Germany), and 5-μm-thick sections were cut with a CM 1850 microtome (Leica, Germany). The sections were collected on Adhesion microscope slides (Citotest, China). Sections were hybridized in a buffer (0.9 M NaCl, 0.02 M Tris-HCl [pH 8.0], 0.01% SDS, 35% formamide) containing probes at a final concentration of 5 ng μl^−1^ for 3 h at 46°C, washed for 30 min at 48°C with washing buffer (0.08 M NaCl, 0.02 M Tris-HCl [pH 8.0], 0.01% SDS, 5 mM EDTA), dipped briefly in Milli-Q water and 96% ethanol, and then air dried. To stain all DNA, sections were covered with 10 μl of a 1-μg ml^−1^ DAPI (4′,6-diamidino-2-phenylindole) solution, incubated for 3 to 10 min, rinsed with Milli-Q water and 96% ethanol, and then air dried. Branchiostegite sections were hybridized using probes Eub338 (67) and DSB706 (15). Observations and imaging were performed using both a fluorescence microscope (DM6000B; Leica, Germany) and a confocal laser scanning microscope (TCS SP5; Leica, Germany).

### Accession number(s).

Metagenomic and metatranscriptomic data were submitted to the Sequence Read Archive (SRA) database of the NCBI under accession numbers SRX4896442 and SRX4896443, respectively. The draft genome of “*Candidatus* Desulfobulbus rimicarensis” was deposited at DDBJ/ENA/GenBank under the accession number RKSL00000000. The version described in this paper is version RKSL01000000.

## Supplementary Material

Supplemental file 1
